# Increased expression of Nrf2 and elevated glucose uptake in pheochromocytoma and paraganglioma with SDHB gene mutation

**DOI:** 10.1186/s12885-022-09415-5

**Published:** 2022-03-18

**Authors:** Takao Kamai, Satoshi Murakami, Kyoko Arai, Daisaku Nishihara, Toshitaka Uematsu, Kazuyuki Ishida, Toshiki Kijima

**Affiliations:** 1 Department of Urology, Dokkyo Medical University, 880 Kitakobayashi Mibu, Tochigi, 321-0293 Japan; 2 Department of Diagnostic Pathology, Dokkyo Medical University, Mibu, Tochigi, Japan

**Keywords:** Pheochromocytoma, Paraganglioma, Nuclear factor E2-related factor 2 (Nrf2), Succinate dehydrogenase (*SDH*), Fumarate hydratase (*FH*), [^18^F]fluorodeoxy-glucose positron emission tomography (^18^F-FDG-PET)

## Abstract

**Background:**

Pheochromocytomas (PCC) and paragangliomas (PGL) are catecholamine-producing neuroendocrine tumors. According to the World Health Organization Classification 2017, all PCC/PGL are considered to have malignant potential. There is growing evidence that PCC/PGL represent a metabolic disease that leads to aerobic glycolysis. Cellular energy metabolism involves both transcription factor nuclear factor erythroid 2-related factor 2 (Nrf2) and succinate dehydrogenase (SDH) subtypes, but the association of these substances with PCC/PGL is largely unknown.

**Methods:**

We investigated SDHB gene mutation and protein expressions for SDHB and Nrf2 in surgical specimens from 29 PCC/PGL. We also assessed preoperative maximum standard glucose uptake (SUVmax) on [^18^F]fluorodeoxy-glucose positron emission tomography and mRNA levels for *Nrf2*.

**Results:**

Among 5 PCC/PGL with a PASS Score ≥ 4 or with a moderately to poorly differentiated type in the GAPP Score, 4 were metastatic and found to be *SDHB* mutants with homogeneous deletion of SDHB protein. *SDHB* mutants showed a higher expression of Nrf2 protein and a higher preoperative SUVmax than non-*SDHB* mutants with a PASS < 4 or a well-differentiated GAPP type. Furthermore, protein expression of Nrf2 was positively associated with preoperative SUVmax. The *Nrf2* mRNA level positively correlated with malignant phenotype, higher expression for Nrf2 protein and SDHB gene mutant, but negatively correlated with expression for SDHB protein. There was also a positive correlation between *Nrf2* mRNA level and SUVmax.

**Conclusion:**

These results suggest that activation of Nrf2 and elevated metabolism play roles in PCC/PGL with malignant potential that have SDHB gene mutation and SDHB deficiency.

## Background

Pheochromocytomas (PCC) and paragangliomas (PGL) are catecholamine-producing neuroendocrine tumors. PCC are rare neuroendocrine tumors that, by definition, arise in the adrenal medulla, and PGL are tumors that arise from sympathetic ganglia in the thorax, abdomen, retroperitoneum, and pelvis and from parasympathetic paraganglia in the head and neck area. Both PCC and PGL are composed of chromaffin cells that derive from the adrenal medulla and sympathetic ganglia [1].

PCC/PGL are rated by two scoring systems, the Pheochromocytoma of the Adrenal gland Scaled Score (PASS) and the Grading system for Pheochromocytoma and Paraganglioma (GAPP); these systems represent the most versatile histopathological index that has been proposed so far for discriminating between benign and malignant tumors [2, 3]. Both the PASS and GAPP grade have a low positive predictive value but a high negative predictive value, suggesting that they are good at ruling out metastatic potential but poor at predicting it [4]. Thus, the World Health Organization (WHO) Classification 2017 specified that all PCC/PGL should be treated as tumors with metastatic potential. The International Classification of Diseases for Oncology 3 subsequently classified these tumors as a category of malignant tumor, as did the 2017 WHO Classification of Tumors of Endocrine Organs [5]. Of clinical relevance is the histopathological classification of PCC/PGL, in which the distinction between benign (non-metastatic) and potentially metastatic tumors on the basis of one or multiple criteria has been virtually impossible with sufficient sensitivity and specificity to be clinically useful. Currently, as discussed in the most recent version of the WHO Classification of Tumors of Endocrine Organs, all PCC and PGL are considered to have malignant potential [5].

Mutations of genes encoding subunits of succinate dehydrogenase (SDH) are predominantly linked to PCC/PGL and are referred to as *SDHx*-mutated PCC/PGL [6, 7]. SDH is a tricarboxylic acid (TCA) cycle enzyme complex consisting of four protein subunits (succinate dehydrogenase A [SDHA], succinate dehydrogenase B [SDHB], succinate dehydrogenase C [SDHC], and succinate dehydrogenase D [SDHD]); it is involved in the mitochondrial electron transport chain and is required for cellular energy metabolism, and altered activity of the SDH is linked with a subset of human cancers in which cell metabolism changes [8, 9]. In PCC/PGL, *SDHB* mutations are highly associated with malignant behavior [10, 11]. A combination of GAPP classification and SDHB immunohistochemistry might be useful for predicting metastasis in these tumors [3]. One of the prominent alterations in PCC/PGL is metabolic reprogramming, which leads to a switch from oxidative phosphorylation to aerobic glycolysis, so these tumors are defined as a metabolic disease [12]. In PCC/PGL, tumor cells grow through the synthesis of macromolecules, including proteins, nucleic acids, lipids, and fatty acids, and increased glucose uptake and glycolysis [13]. Cancer cells require a constant supply of nutrients to maintain energy metabolism and protein synthesis for rapid proliferation, and this elevated demand may be met by increasing nutrient availability through vasculogenesis and/or enhancing cellular uptake via the upregulation of certain transporters [14]. In particular, as a result of metabolic reprogramming, cancer cells depend on aerobic glycolysis to provide energy, a characteristic known as the Warburg effect [15]. Uptake of [^18^F]fluorodeoxy-glucose (^18^F-FDG) can be used to measure glucose consumption by aerobic and anaerobic glycolysis, and ^18^F-FDG-positron emission tomography (^18^F-FDG-PET) exploits the high glucose uptake/utilization by tumor cells, providing a clinically powerful tool for metabolic evaluation of many cancers [16, 17]. In clinical practice, ^18^F-FDG-PET can be used for cancer staging and measurement of therapeutic response by assessing cellular glucose metabolism; the most common semiquantitative parameter for cellular glucose metabolism is the maximum standard glucose uptake value (SUVmax) [16, 17]. It has been reported that *SDHB* mutated tumors are more prone to become metastatic and show high glucose uptake [11, 18]. However, the assessment of metabolic activity by ^18^F-FDG-PET in PCC/PGL has yet to be fully studied, and few studies have investigated molecular mechanisms in the relationship between PASS/GAPP and *SDHB* gene mutations from a metabolic perspective.

There is growing evidence that the transcription factor nuclear factor erythroid 2-related factor 2 (Nrf2) plays a vital role in tumor proliferation by promoting metabolic activity and regulating antioxidant pathways for detoxifying reactive oxygen species (ROS), a major cellular defense mechanism. Although inhibition of Nrf2 signaling prevent carcinogenesis and tumor progression in normal tissues, activation of Nrf2 signaling supports malignant cells by promoting chemoresistance and proliferation [19, 20]. In addition, research recently showed that constitutive activation of Nrf2 promotes metabolic activity that supports cell proliferation and that increased expression of Nrf2 in tumors is associated with a poor prognosis [21, 22]. Thus, constitutive Nrf2 activation appears to be involved in the development and progression of various human cancers. Although the question of whether the Nrf2 pathway is activate or not in SDHB related tumors is interesting and debated [23, 24], the Nrf2 pathway in in PCC/PGL is poorly convincing.

In the current study, we analyzed Nrf2 expression and ^18^F-FDG uptake in PCC/PGL and also evaluated *SDHB* gene mutation and SDHB expression in these tumors. Such observations provide information about the biological significance of metabolic reprogramming in PCC/PGL with malignant potential.

## Methods

### Patients

This was a retrospective study of 29 patients (18 men and 11 women; median age, 47 years; range, 27–71 years) with histopathologically diagnosed PCC (*n* = 21) or PGL (*n* = 8), who underwent surgical resection for the tumor at Dokkyo Medical University Hospital between 2011 and 2020. For staging, all patients underwent preoperative computed tomography (CT) and/or magnetic resonance imaging (MRI), in addition to a scintigraphic study using metaiodobenzylguanidine (MIBG) scan; MIBG is a norepinephrine analog and is labeled with iodine-123 for the scan. Furthermore, all patients also underwent whole-body imaging with a combined ^18^F-FDG-PET/CT scanner (^18^F-FDG-PET/CT) (Biograph, Sensation 16, Siemens Systems). Data processing was performed as reported previously [25]. The pretreatment maximum standard glucose uptake (SUVmax) value on ^18^F-FDG-PET was defined as the baseline SUVmax. Twenty-eight patients underwent surgical resection for primary PCC/PGL and metastases, but one patient underwent resection for a paraganglioma and received systemic therapy for bone metastasis. The postoperative follow-up period ranged from 7 to 108 months, with a median of 35 months. For detection of metastatic disease, CT and/or MRI was performed every 2 to 4 months. Final assessment was done by review of the medical records in November 2021.

The cellular classification used in this study was based on the PASS and GAPP [2, 3]. The PASS was used to divide PCC/PGL into two groups: tumors with a PASS less than 4, and tumors with a PASS greater than or equal to 4 [2]. In addition, the GAPP score was used to divide them into 3 groups: 1 to 2 points, well differentiated type; 3 to 6 points, moderately differentiated type; and 7 to 10 points, poorly differentiated type [3]. Thus, in this study, we classified tumors into tumor groups; benign tumors with a PASS below 4 and a GAPP grade of well-differentiated type and malignant tumors with a PASS greater than or equal to 4 or a GAPP grade of moderately to poorly differentiated type.

### DNA extraction

Frozen tumor samples were ground to a powder in liquid nitrogen, and 30 to 50 mg of the powder was used for DNA extraction with an AllPrep kit (Qiagen). DNA was quantified, and its purity was assessed with a NanoDrop ND-1000 spectrophotometer (Labtech). Blood DNA was extracted from leukocytes according to the standard protocols.

### Next-generation sequencing

Next-generation sequencing was performed to detect single nucleotide variants (SNVs), short insertions, and deletions (indels). We investigated mutations of the *SDHA, SDHB, SDHC, SDHD, fumarate hydratase (FH)* and rearranged during transfection *(RET)* genes by sequencing the coding exons and intron flanking regions in tumor specimens, as described previously [26]. The custom primers for these regions were designed with Ampliseq Designer (Life Technologies). Library construction and sequencing were performed with an Ion AmpliSeq Library Kit 2.0, Ion PGM IC 200 kit, and Ion PGM (Life Technologies), according to the manufacturer's instructions. Sequencing data were analyzed with Torrent Suite, and variant call was conducted with Torrent Variant Caller, Ion Reporter (v.5.1.0). Ion AmpliSeq panels cover broad research areas for germline analysis, including genes recommended by the American College of Medical Genetics and Genomics [27]. Then, the accuracy of the Ion Torrent sequencer platform in detecting *SDHB* gene mutations was confirmed according to a previously published method [28].

### Data analysis

Data were analyzed according to a previous report [26]. Briefly, after each sequencing reaction, raw data were analyzed by using Torrent Suite version 4.2.1 for signal processing, base calling, quality score assignment, adapter trimming, mapping to Genome Reference Consortium Human Build 37/Human Genome version 19, assessment of mapping quality, and variant calling. After completion of primary data analysis, a list of the sequence variants detected (SNVs and indels) was compiled in a variant call file format and presented via the web-based user interface. The results of mapping and variant calling were visualized by using Integrative Genome viewer (Broad Institute).

### Immunohistochemistry

Surgically resected tumor tissue specimens were sectioned at a thickness of 4 μm, fixed in formalin and embedded in paraffin. Immunohistochemical staining of tumor specimens from the 29 patients was performed with mouse monoclonal antibodies for Nrf2 (Abcam, ab-62352), SDHA (Abcam, 2E3GC12FB2AE2) and SDHB (Abcam, 21A11AE7) [26]. The tumors were divided into two groups: a low expression group, in which fewer tumor cells were positive for anti-Nrf2, anti-SDHA, and anti-SDHB antibodies (< 30% of all tumor cells were positive), and a high expression group, in which more tumor cells were positive for these antibodies (≥ 30% of all tumor cells were positive) [26].

### Real-time reverse transcription-polymerase chain reaction (Real-time RT-PCR) assay

A real-time RT-PCR assay was performed on a 25 mL reaction mixture containing 20 ng of sample cDNA, 100 nM sense primer, 100 nM anti-senseprimer, and 12.5 mL of SYBR Green PCR Master Mix (Applied Biosystems). The primers were employed to amplify the indicated genes in the primary tumors, according to those described previously [29]. The PCR was carried out for 50 cycles of 95 °C for 15 s and 60 °C for 1 min. To normalize the amplified products in each sample, we used beta actin as a quantitative internal control. A standard curve for each mRNA expression was generated using five-fold dilutions of a control RNA sample (25x, 5x, 1x, 0.2x, 0.04x). The mRNA expression levels of *Nrf2* gene were presented as a ratio to that of beta actin, and the relative expression levels were calculated. The three samples of the resected tissues were analyzed for the mean values from the real-time RT-PCR data.

### Statistical analysis

Pearson’s χ2 test for contingency tables was employed to assess the association of Nrf2 protein expression with the expression of SDHA and SDHB proteins, as well as the relationship between *Nrf2* protein expression and PASS and GAPP Scores. The Mann–Whitney U test was used to compare SUVmax or mRNA level for Nrf2 with PASS score, GAPP score, and protein expressions for SDHA, SDHB and Nrf2. Spearman’s rank correlation coefficient analysis was performed to determine the relations between mRNA level for *Nrf2* and SUVmax. Analyses were performed with commercially available software, and *p* < 0.05 was considered to indicate significance.

The study was conducted in accordance with the Declaration of Helsinki and was approved by the ethics review board of Dokkyo Medical University Hospital. Every patient signed an informed consent form that was approved by our institutional Committee on Human Rights in Research.

## Results

### Classification by PASS Score and GAPP Score

The number of patients in each of the PASS and GAPP score subgroups is shown in Table 1. In PASS score, benign tumor with a PASS below 4 was 23 cases and malignant tumor with a PASS greater than or equal to 4 was 6 cases. By GAPP score, benign tumor with a GAPP grade of well-differentiated type was 18 cases and malignant tumor with a GAPP grade of moderately to poorly differentiated type was 11 cases. Thus, benign tumors with a PASS below 4 and a GAPP grade of well-differentiated type was 18 cases and malignant tumors with a PASS greater than or equal to 4 or a GAPP grade of moderately to poorly differentiated type was 11 cases. Four metastatic tumors had tumors with a GAPP grade of poorly differentiated type. Among these four patients, three patients had been undergone complete resection of primary and metastatic lesions and alive with no evidence of disease, while one patient underwent resection for a primary tumor and received systemic therapy for bone metastasis and alive with disease.

**Table 1:** Classification of pheochromocytoma and paraganglioma

	PASS		GAPP			Metastasis	
	Benign	Maligant	Benign	Malignant		GAPP with poor differentiation	
	< 4	≥ 4	well	moderate	poor	M0	M1
Pheochromocytoma (*n* = 21)	20	1	18	3	0	21	0
Paraganglioma (*n* = 8)	3	5	0	4	4	4	4

**Fig. 1:**
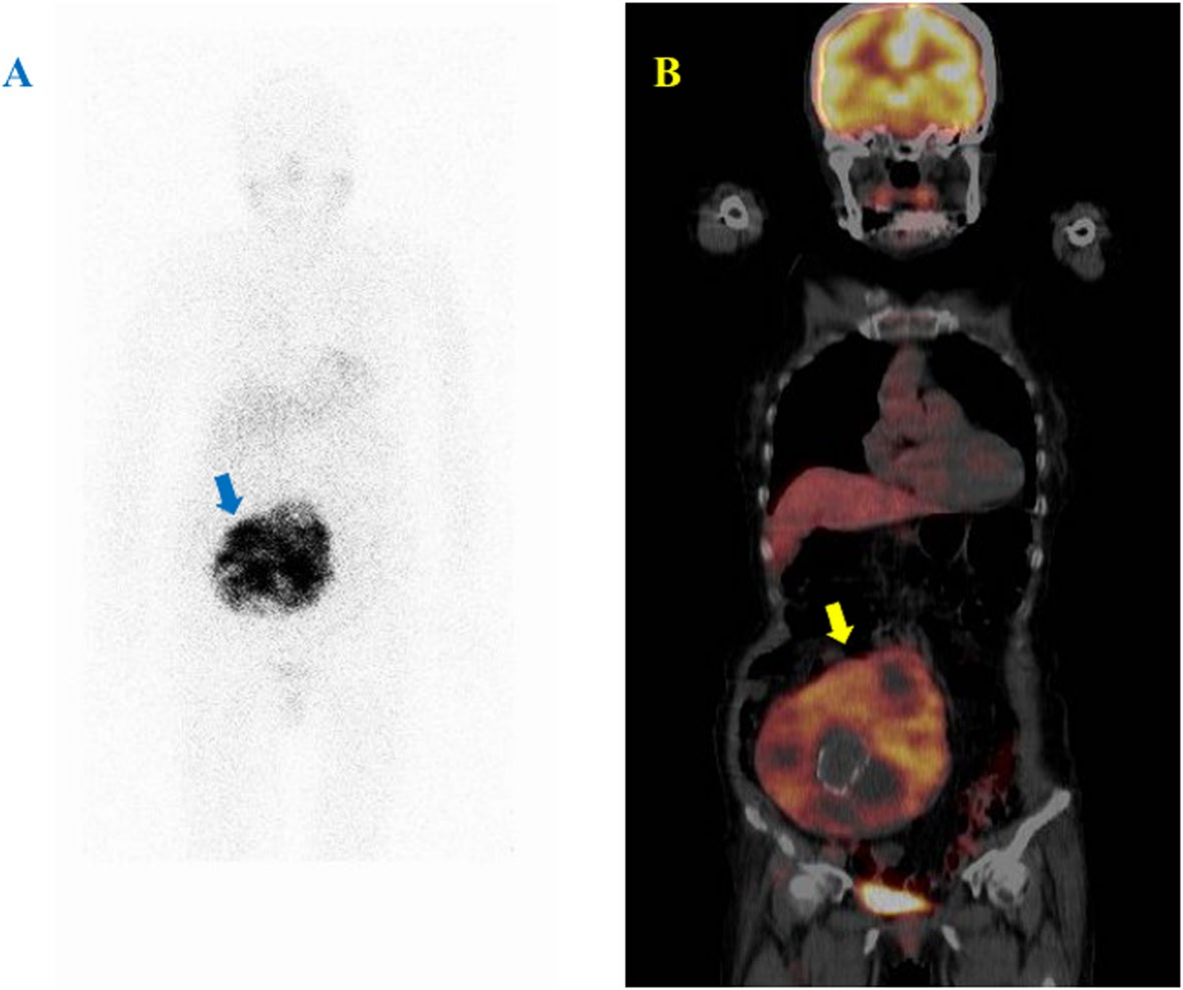
^18^F-FDG-PET and MIBG scintigraphy in paraganglioma without SDHB gene mutation. Sixty-one y.o. female of paraganglioma without SDHB gene mutation. (**A**) ^123^I-MIBG scintigraphy showed positive accumulation in right retroperitoneal to pelvic cavity. (**B**) ^18^F-FDG-PET showed SUVmax:5.0 in the mass. The tumor had PASS Score with < 4 and GAPP Score with well differentiation

**Fig. 2:**
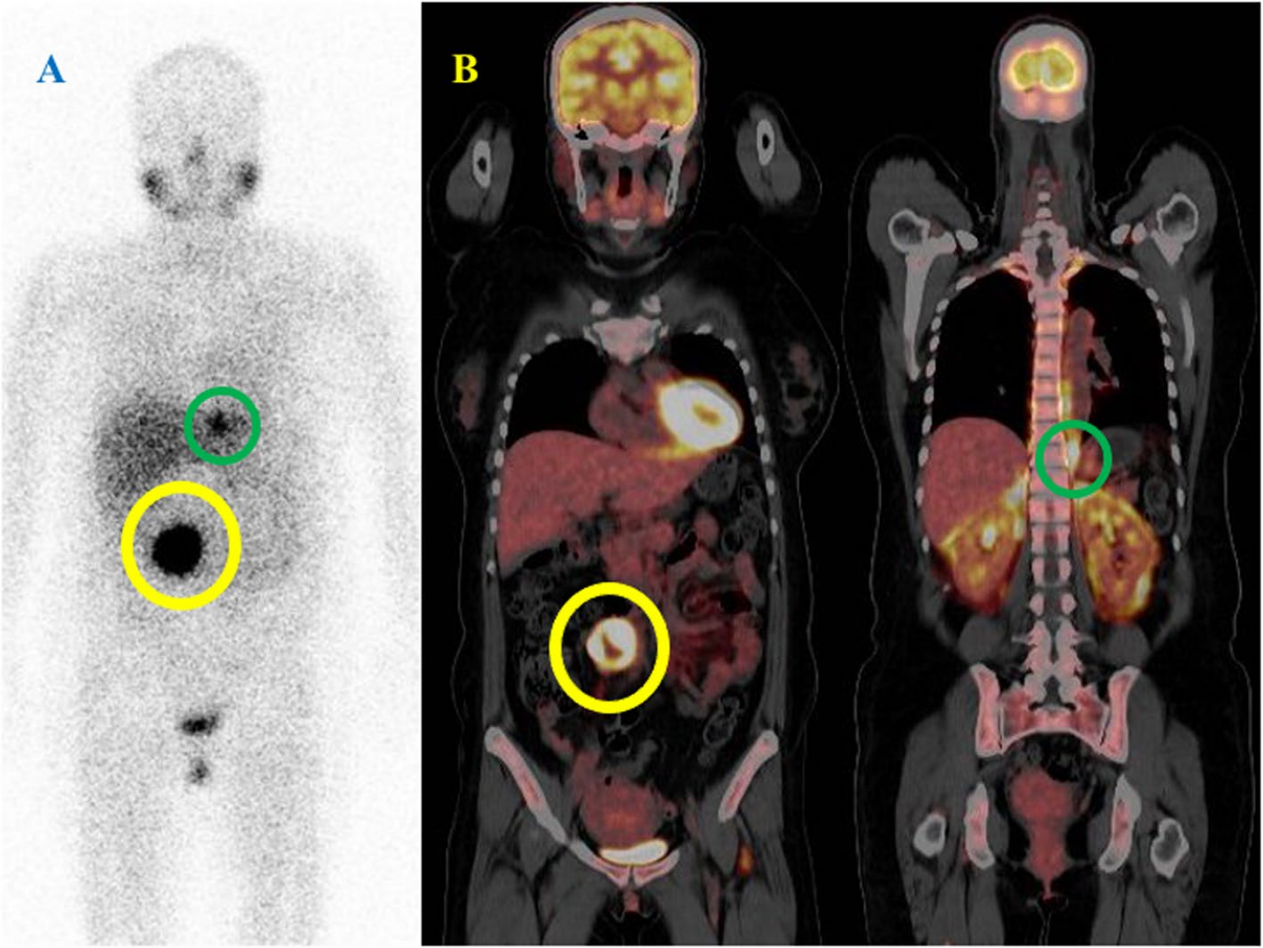
^18^F-FDG-PET and MIBG scintigraphy in paraganglioma with SDHB gene mutation. Thirty-seven y.o. female of paraganglioma with SDHB gene mutation. (**A**) ^123^I-MIBG scintigraphy showed positive accumulation in right retroperitoneal cavity (yellow circle) and left retroperitoneal cavity (green circle). (**B**) ^18^F-FDG-PET showed SUVmax:24.2 in right retroperitoneal cavity (yellow circle) and SUVmax:17.8 in left retroperitoneal cavity (green circle). Both tumors had PASS Score ≥ 4 and GAPP Score with poorly differentiation

The mean SUVmax calculated by ^18^F-FDG-PET was 7.3 ± 3.6. Tumors with a PASS greater than or equal to 4 and/or a GAPP grade of moderately to poorly differentiated type had a significantly higher SUVmax than those with a PASS of less than 4 and/or a GAPP grade of well-differentiated type (13.2 ± 9.0 vs 4.8 ± 0.7, respectively; *p* < 0.0001; see Table 2 and Figs. 1 and 2).

**Table 2:** Relationship between gene mutation of SDHB, protein expression of SDHB and Nrf2, and FDG-PET

Pheochromocytoma (*n* = 21) / Paraganglioma (*n* = 8)	SDHB gene mutation		*P* value	SDHB expression		*P* value	Nrf2		*P* value	SUVmax (mean ± S.D)	*P* value
	No (*n* = 25)	Yes (*n* = 4)		Lower (Non to Low) (*n* = 5)	Higher (Moderate to High) (*n* = 24)		Lower (Non to Low) (*n* = 21)	HIgher (Moderate to High) (*n* = 8)			
PASS < 4 or GAPP with well differentiation (*n* = 18)	18	0	< 0.0001	0	18	< 0.0001	17	1	0.0011	4.8 ± 0.7	0.0026
PASS ≥ 4 or GAPP with moderately to poorly differentiation (*n* = 11)	7	4		5	6		4	7		13.2 ± 9.0	
Metastasis											
No:M0 (*n* = 25)	25	0	< 0.0001	1	24	< 0.0001	21	4	0.0029	5.9 ± 2.4	0.0019
Yes:M1 (*n* = 4)	0	4		4	0		0	4		20.5 ± 8.9	
SDHB gene mutation											
No (*n* = 25)				1	24	< 0.0001	21	4	0.0005	5.9 ± 2.4	0.0019
Yes (*n* = 4)				4	0		0	4		20.5 ± 8.9	
SDHB expression											
Negative (Non—Low) *(n* = 5)							1	4	0.0047	5.3 ± 1.0	0.0018
Positive (Moderate—High) (*n* = 24)							20	4		18.9 ± 8.4	
Nrf2 expression											
Negative (Non—Low) (*n* = 21)										5.9 ± 2.5	0.0039
Positive (Moderate—High) (*n* = 8)										17.8 ± 9.8

### Next-generation sequencing

Targeted next-generation sequencing of coding exons for *SDHB* gene revealed four mutations associated with amino acid sequence variation in four out of 29 PCC/PGL with metastatic lesions. Four PCC/PGL with metastatic lesions with a PASS greater than 4 or a GAPP grade of poorly differentiated type had the *SDHB* gene mutations: exon3, c.201-2A > C (rs878854574); exon 3, c.268C > T, p.Arg90Ter, (rs74315366); exon 3, c.274 T > C, p.Ser92Pro, (rs1553178041); and exon 4, c.293G > A, p.Cys98Tyr (rs1553177768). On the other hand, among 25 PCC/PGL with a PASS below 4 or a GAPP grade of well to moderately differentiated type, 15 tumors had a synonymous SDHB gene mutation without amino acid sequence variation: exon 1, c.18C > A, *p*.( =) (rs2746462). Furthermore, among such 15 synonymous mutation of SDHB gene, 9 tumor had RET gene mutation: exon 11, c.1901G > T, p.Cys634Phe (rs75996173): exon 11, c.2071G > A, p.Gly691Ser (rs1799939): or exon 11, c.1925 T > C, p.Val642Ala (rs766962871). The tumors had no mutation of *SDHA*, *SDHC*, *SHDH* and *FH* genes.

### Immunohistochemistry for SDHA, SDHB and Nrf2 and its relation to SUVmax

SDHA was expressed in all tumor cells, regardless of whether the cells were positive or negative for *SDHB* gene mutations.

**Fig. 3:**
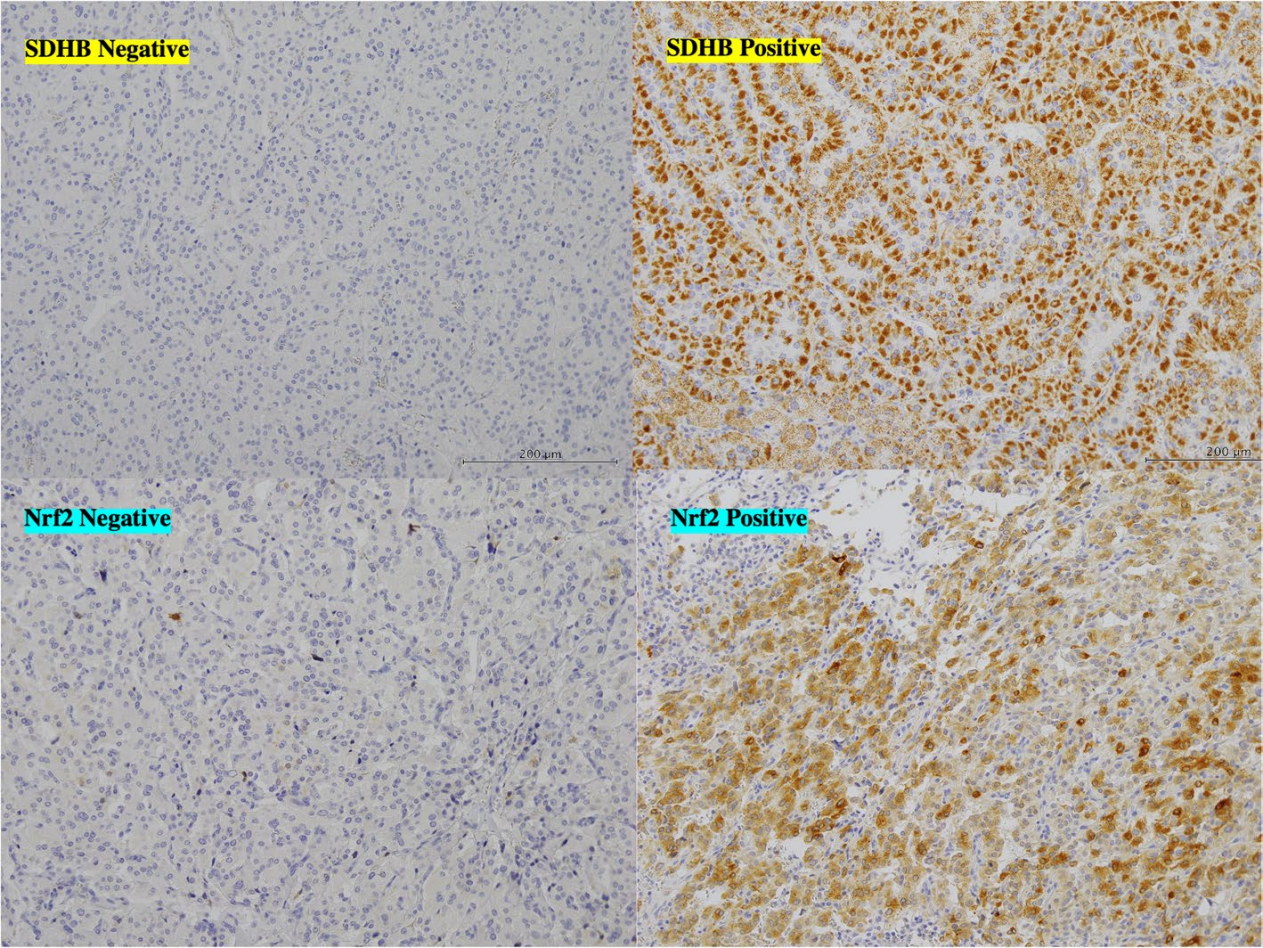
Immunohistochemistry for SDHB and Nrf2. Immunohistochemical staining using anti-SDHB antibody (upper panel) (× 200 magnification). Upper light panel shows negative staining in many of the cancer cells. Upper left panel shows intensely staining in most of the cancer cells, displaying high SDHB protein levels. Lower panels show immunohistochemical staining using anti-Nrf2 antibody (× 200 magnification). Lower light panel shows negative staining in many of the cancer cells. Lower left panel shows intensely staining in most of the cancer cells, displaying high Nrf2 protein levels

**Fig. 4:**
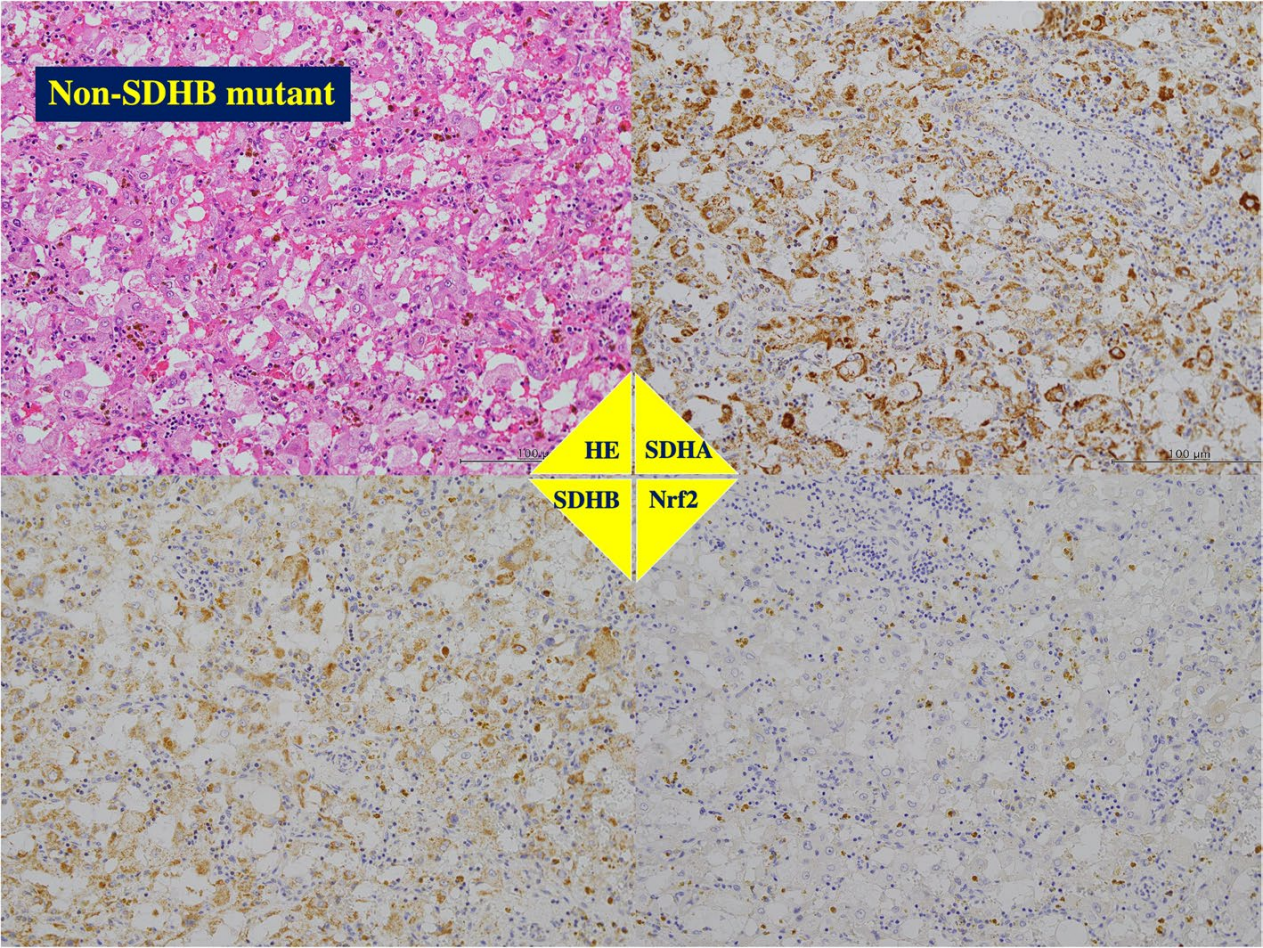
Immunohistochemistry of paraganglioma without SDHB gene mutation. The same patient presented in Fig. 1. The tumor had PASS Score with < 4 and GAPP Score with well differentiation, and showed intense staining for anti-SDHA, anti-SDHB and anti-Nrf2 antibodies. The patient is alive with disease-free for 97 months

**Fig. 5:**
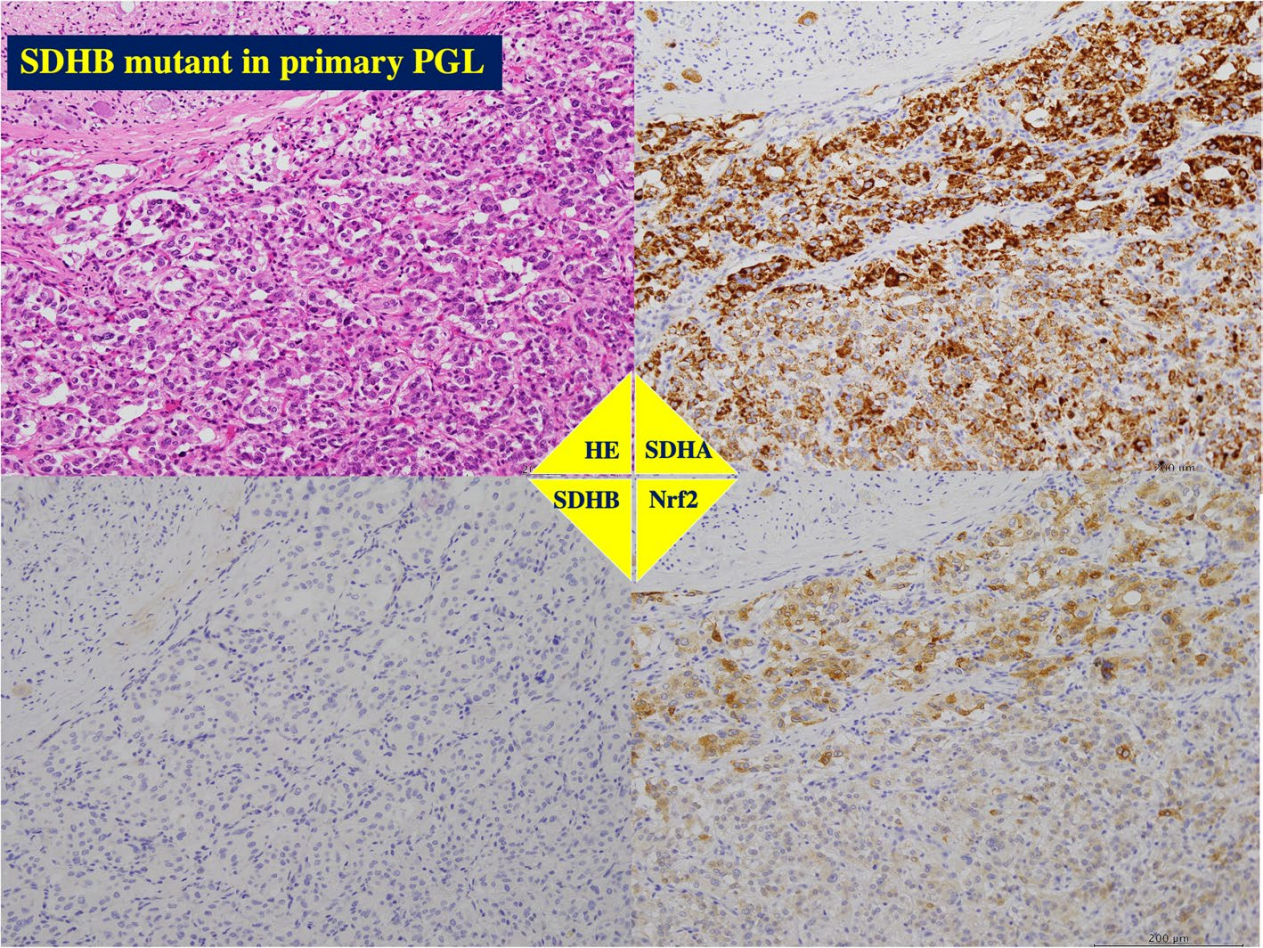
Immunohistochemistry of paraganglioma with SDHB gene mutation. The same patient presented in Fig. 2. The tumor in right retroperitoneal cavity had PASS Score ≥ 4 and GAPP Score with moderately to poorly differentiation, and showed the positive reaction for anti-SDHA and anti-Nrf2 antibodies, but not for anti-SDHB antibody. The patient is alive with disease-free for 57 months

Primary tumors without *SDHB* gene mutation had positive immunostaining for both anti-SDHA and anti-SDHB antibodies, regardless of the expression levels of Nrf2 (Figs. 3 and 4). On the other hand, these *SDHB*-mutant tumors showed no immunostaining with anti-SDHB antibody (*p* < 0.0001) and higher staining anti-Nrf2 antibody (*p* = 0.0005, Table 2 and Figs. 3 and 5).

The malignant phenotype with a PASS greater than or equal to 4 or a GAPP grade of moderately to poorly differentiated type was associated with *SDHB* gene mutant (*p* < 0.0011), lower SDHB expression (*p* < 0.0001), higher Nrf2 expression (*p* = 0.0011) and a higher SUVmax (*p* = 0.0026, Table 2). The metastatic phenotype was related with *SDHB* gene mutant (*p* < 0.0011), lower SDHB expression (*p* < 0.0001), higher Nrf2 expression (*p* = 0.0029) and a higher SUVmax (*p* = 0.0019, Table 2).

**Fig. 6:**
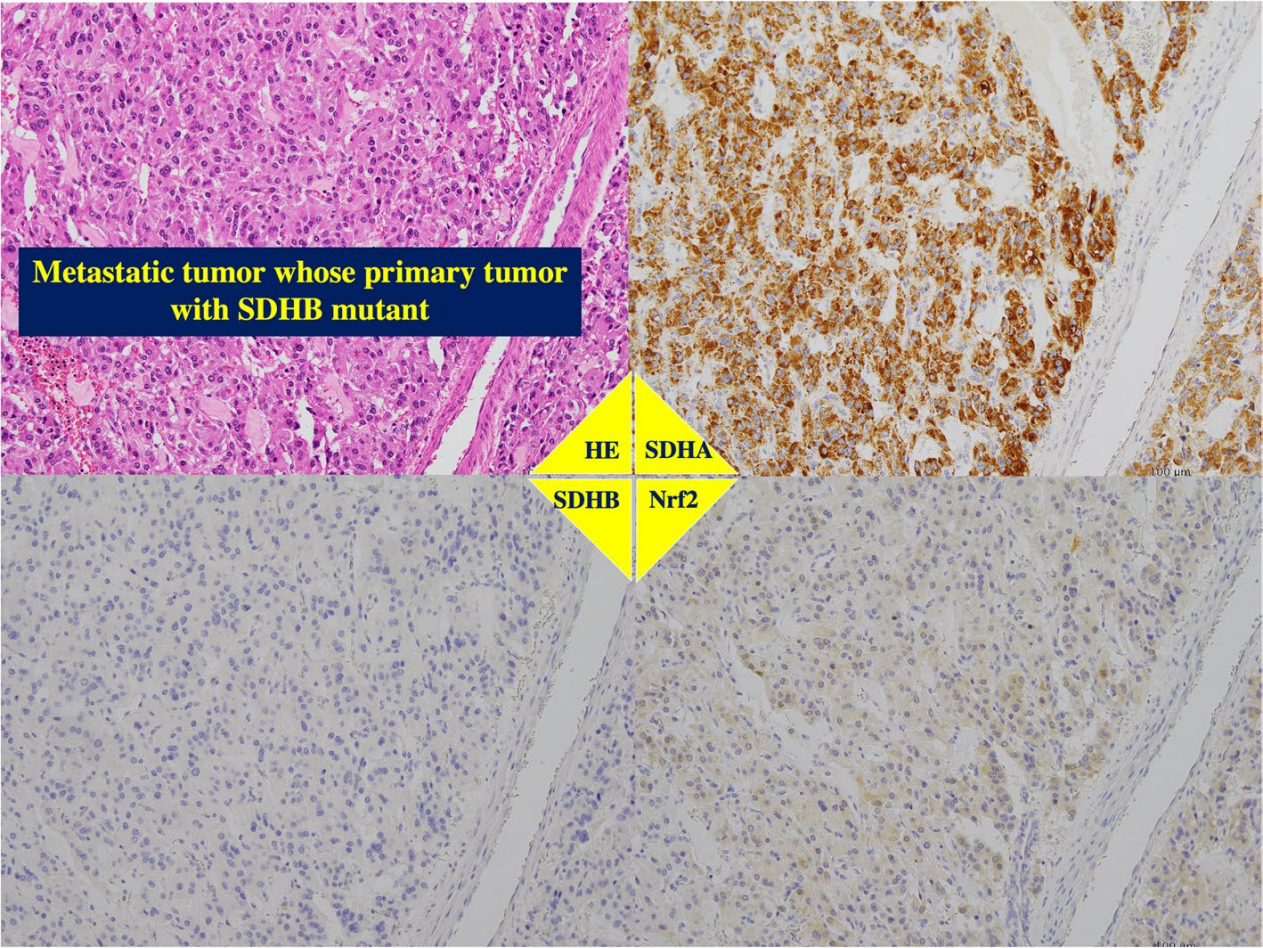
Immunohistochemistry of metastatic tumor of the primary paraganglioma with SDHB gene mutation. The same patient presented in Figs. 2 and 4. The tumor in left retroperitoneal cavity had PASS Score ≥ 4 and GAPP Score with moderately to poorly differentiation. As well as the paraganglioma in right retroperitoneal cavity, this tumor showed the positive staining using anti-SDHA and anti-Nrf2 antibodies, but not using anti-SDHB antibody

The metastatic lesions from the *SDHB* mutant primary tumors showed positive staining with anti-SDHA and anti-Nrf2 antibodies but not with anti-SDHB antibody (Fig. 6).

The expression of SDHB was positively associated with Nrf2 proteins (*p* = 0.0047, Table 2). Furthermore, a higher expression of Nrf2 in the primary tumor was significantly associated with higher SUVmax (*p* = 0.0039) (Table 2).

### RT-PCR assay for Nrf2 mRNA

**Fig. 7:**
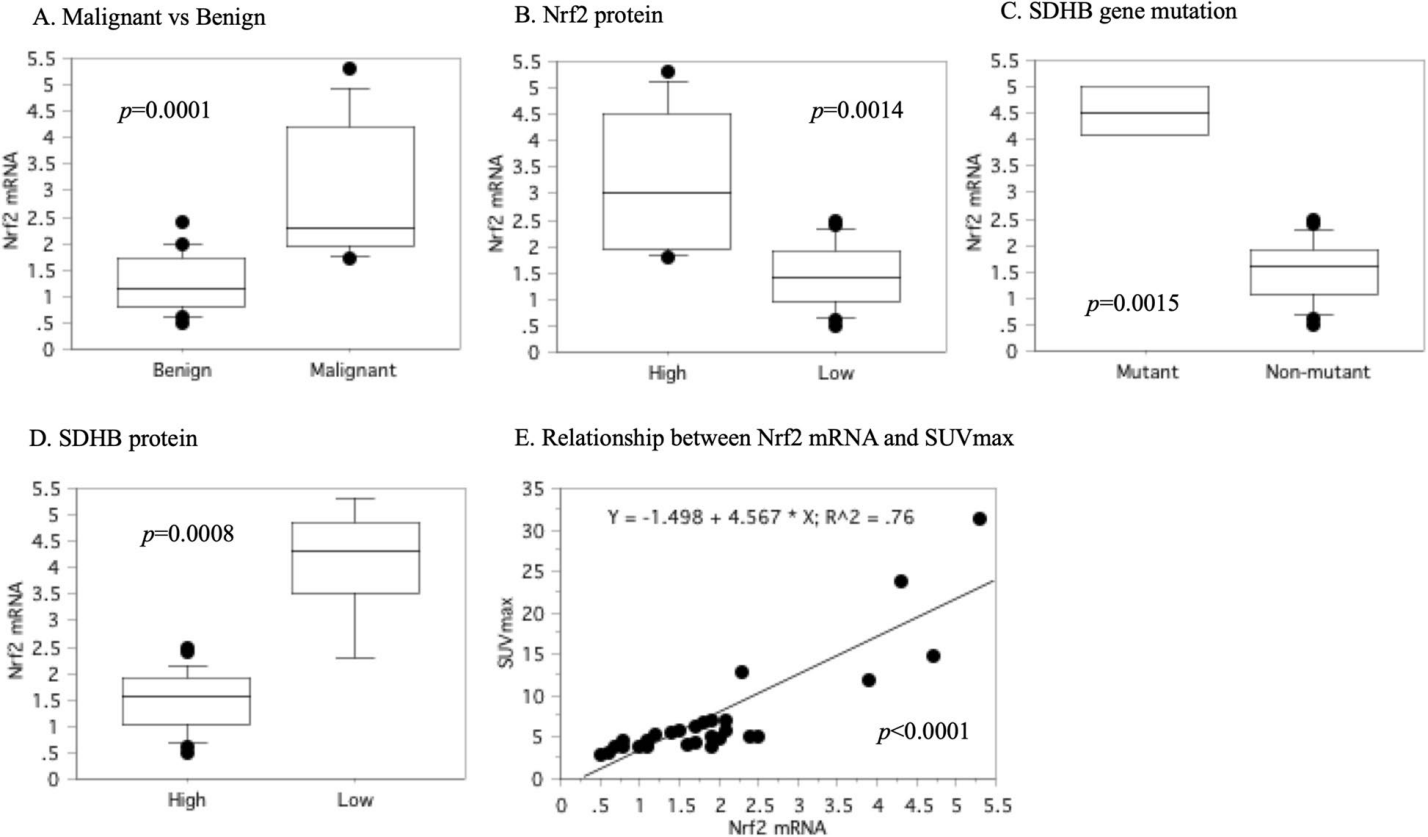
A real-time RT-PCR. Increased Nrf2 mRNA level was associated with malignant phenotype (**A**), higher expression for Nrf2 protein (**B**), and *SDHB* gene mutant (**C**), but with lower expression for SDHB protein (**D**). There was a positive correlation between *Nrf2* mRNA level and SUVmax (**E**)

The *Nrf2* mRNA level positively correlated with malignant phenotype (*p* = 0.0001, Fig. 7A), expression for Nrf2 protein (*p* = 0.0014, Fig. 7B), and *SDHB* gene mutant (*p* = 0.0015, Fig. 7C), but negatively related with expression for SDHB protein (*p* = 0.0014, Fig. 7D). There was also a positive correlation between *Nrf2* mRNA level and SUVmax (r^2^ = 0.76, *p* < 0.0001, Fig. 7E).

## Discussion

In the present study in patients with PCC/PGL, we investigated the association of Nrf2 and SUVmax with the PASS and GAPP score and *SDHB* gene mutations from a metabolic perspective. The malignant tumors with a higher PASS (≥ 4) and/or a GAPP grade of moderately to poorly differentiated type had an increased expression of *Nrf2* mRNA and protein expressions, an elevated SUVmax on ^18^F-FDG-PET, and *SDHB* gene mutation. Furthermore, the tumors with *SDHB* gene mutation were associated with metastatic phenotype, homogeneous deletion of SDHB, increased *Nrf2* mRNA and protein expressions, and higher SUVmax. Higher expressions of *Nrf2* mRNA and protein were also associated with a higher SUVmax. These observations suggest that *Nrf2* mRNA and protein expressions is related to malignant and metastatic potential and disease progression in PCC/PGL, in particular in case of *SDHB* gene mutation with SDHB deficiency.

Recently, the relationship between the clinical features of in PCC and PGL and their genotyping has become clearer. The development of gene analysis in recent years has enabled researchers to clarify that the frequency of inherited PCC/PGL, which was conventionally thought to be about 10%, is actually about 30% to 40% [30]. Furthermore, both germline and somatic mutations have been reported. Currently, 35% to 40% of PCC/PGL are assumed to carry germline mutations [30]. These mutations are found in more than 20 different genes but functionally clustered into two major clusters: cluster 1, which is characterized by the pseudohypoxia pathway and includes, among others, PCC/PGL with von Hippel-Lindau (*VHL*) and *SDHx* mutations; and cluster 2, which is characterized by activation of kinase signaling pathways and includes, among others, PCC/PGL with *RET* and neurofibromatosis type 1 (*NF1*) gene mutations [31–33]. Among those gene mutations, mutation of *SDHx* is predominantly linked to PCC/PGL [6, 7]. The tumor cells of *SDHB*-, *SDHC*-, and *SDHD*-mutated tumors were reported to show absence of SDHB immunostaining, whereas those of non-*SDH* mutated tumors showed strong SDHB immunostaining, and SDHB immunohistochemistry was found to have 100% sensitivity and 84% specificity in detecting mutations [34]. Furthermore, a combination of GAPP classification and SDHB immunohistochemistry might be useful for predicting metastasis in these tumors [3]. In the present study, PCC/PGL with *SDHB* mutant showed SDHB deficiency and a higher risk of metastasis, indicating that additional signaling is involved in the progression of these diseases. To date, few studies have investigated the relationship of PASS/GAPP or *SDHB* gene mutations with molecular mechanisms from a metabolic perspective.

In addition to the well-documented cancer-preventive antioxidant function of Nrf2 signaling [35], many lines of evidence indicate that Nrf2 promotes various metabolic pathways and cell proliferation in cancers [19–22]. As mentioned above, tumor cells typically switch to glycolysis to generate adenosine triphosphate, and the increased glucose uptake associated with this elevated glycolysis is a key change in cancer cells [14, 15]. It has been known that SDHB mutated tumors are more prone to become metastatic, lose SDHB expression and high glucose uptake [11, 18]. In the present study, PCC/PGL with *SDHB* mutant had higher SUVmax, a higher SUVmax of the primary tumor was associated with metastatic potential, and a higher expression of Nrf2 was significantly associated with a higher SUVmax. It has been reported that antioxidant responses were increased notably through the upregulation of Nrf2 [23]. Consistently with these observation [11, 18, 23], we also found that PCC/PGL were associated with homogeneous deletion of SDHB, increased *Nrf2* mRNA and protein expressions, and higher SUVmax, in particular in case of *SDHB* gene mutation. We recently reported that elevated expression of Nrf2 protein was associated with cancer progression in renal cell carcinoma (RCC) [36], and also reported that increased metabolic activity assessed by ^18^F-FDG-PET was related with elevated expression of Nrf2 protein and cancer progression in upper urinary tract cancer [37]. Taken together, these findings indicate that activation of Nrf2 might be associated with increased glucose uptake and that acceleration of glycolysis might be associated with more malignant behavior in PCC/PGL.

Research has shown that immunohistochemical staining for *S*-(2-succinyl)cysteine (2SC) and FH enable the detection of *FH* gene aberrations in RCC [38]. 2SC is produced by a Michael addition reaction between the TCA cycle intermediate, fumarate, and thiol groups in proteins. This process is known as protein succination which leads to irreversible inactivation of the protein [39]. In the present study, the tumor cells of SDHB-deficient tumors with *SDHB* gene mutation showed higher expression of Nrf2 than the tumors without this mutation, indicating that a sustained activation of Nrf2 has a role in tumorigenesis of PCC/PGL with *SDHB* gene mutations. On the other hand, succinate production was increased in SDHB-deficient tumors. As succinate is known to inhibit prolyl hydroxylase which degrades hypoxia-inducible factor (HIF) under normoxic conditions, SDHB-deficiency in tumors leads to accumulation of HIF [8, 9]. Generation of ROS by *SDHx* mutations also leads to inhibition of prolyl hydroxylase, suggesting that increased ROS production mediates pseudo hypoxia in tumors with *SDHx* mutation and in hereditary leiomyomatosis and renal cell carcinoma in which FH-associated changes might develop in an HIF-dependent manner and with an Nrf2-associated antioxidant phenotype [40–43]. As SDHx and FH are adjacent enzymes in the TCA cycle and their deficiency is presumed to cause similar energy abnormalities and pathological conditions in such PCC/PGL and RCC [12, 13, 40, 41]. Further study should examine the expression of 2SC to assess the roles of the Nrf2 pathway as well as the HIF pathway in tumorigenesis of PCC/PGL with *SDHB* mutation. Although we investigated gene mutations of only *SDHA, SDHB, SDHC, SDHD, FH* and *RET* genes in this cohort, in comparison to *SDHB* gene mutant with amino acid sequence variation with metastatic PCC/PGL with a PASS greater than 4 or a GAPP grade of poorly differentiated type, some PCC/PGL with a PASS below 4 or a GAPP grade of well to moderately differentiated types had a synonymous *SDHB* gene mutation without amino acid sequence variation and *RET* gene mutation, indicating there might be different signaling between metastatic and non-metastatic PCC/PGL. Given that a large number of susceptibility genes are implicated in tumorigenesis and progression of PCC/PGL and approximately 20% of patients diagnosed with a PCC/PGL carry a germline mutation, we should check, at least, the basic set of genes more frequently involved in the disease development and the germline status of the variants [44], which might be lead to more in detail understandings of four molecularly distinguishable groups: a pseudohypoxia subtype (cluster 1), a kinase signaling subtype (cluster 2), a Wnt pathway altered subtype, and a cortical admixture subtype.

The present study had several limitations, including its retrospective design, the relatively small number of participants, and a follow-up period that was too short to allow definite conclusions to be reached. Furthermore, we did not study the mutation of *Nrf2* gene in PCC/PGL. The association of increased expression of Nrf2 and decreased expression of SDHB with glucose uptake should also be studied in the future. Thus, so far, the association of *SDHx* gene mutation with the Nrf2 pathway in PCC/PGL has not been fully elucidated. We showed that four PCC/PGL with SDHB mutation showed SDHB deficiency and that these tumors were scored as moderately to poorly differentiated type by GAPP, had an increased Nrf2 protein expression, and an elevated SUVmax and were metastatic. Furthermore, PCC/PGL may be found to have metastases several years to several decades after complete resection of the primary tumor. Therefore, PCC/PGL with a PASS greater than or equal to 4 or a moderately to poorly differentiated type according to the GAPP score, and in particular those with an increased Nrf2 and elevated SUVmax, may be likely to metastasize in the future, even if no metastatic lesion was present at surgery. These findings suggest that we need to perform long-term active surveillance in patients with such PCC/PGL and that the functions and mutations of both *SDHx* and *Nrf2* should be evaluated in more detail to support the development of new treatment options for PCC/PGL. Our findings need to be confirmed by further investigations, preferably large-scale prospective controlled trials.

## Data Availability

All data generated or analyzed during this study are included in this published article.

## References

[1] Lenders JW, Duh QY, Eisenhofer G, Gimenez-Roqueplo AP, Grebe SK, Murad MH, Naruse M, Pacak K, Young WF, Endocrine Society (2014). Pheochromocytoma and paraganglioma: an endocrine society clinical practice guideline. J Clin Endocrinol Metab.

[2] Thompson LD (2002). Pheochromocytoma of the Adrenal Gland Scaled Score (PASS) to Separate Benign from Malignant Neoplasms: A Clinicopathologic and Immunophenotypic Study of 100 Cases. Am J Surg Pathol.

[3] Kimura N, Takayanagi R, Takizawa N, Itagaki E, Katabami T, Kakoi N, Rakugi  H, Ikeda Y, Tanabe A, Nigawara T, Ito S, Kimura I, Naruse M (2014). Phaeochromocytoma Study Group in Japan. Pathological grading for predicting metastasis in phaeochromocytoma and paraganglioma. Endocr Relat Cancer.

[4] Koh JM, Ahn SH, Kim H, Kim BJ, Sung TY, Kim YH, Hong SJ, Song DE, Lee SH (2017). Validation of Pathological Grading Systems for Predicting Metastatic Potential in Pheochromocytoma and Paraganglioma. PLoS ONE..

[5] Lloyd RV, Osamura RY, Kloppel G, Rosai J (2017). WHO Classification of Tumours of Endocrine System.

[6] Barletta JA, Hornick JL (2012). Succinate dehydrogenase-deficient tumors: diagnostic advances and clinical implications. Adv Anat Pathol.

[7] Gill AJ (2012). Succinate dehydrogenase (SDH) and mitochondrial driven neoplasia. Pathology.

[8] Eng C, Kiuru M, Fernandez MJ, Aaltonen LA (2003). A role for mitochondrial enzymes in inherited neoplasia and beyond. Nat Rev Cancer.

[9] Gottlieb E, Tomlinson IP (2005). Mitochondrial tumor suppressors: A genetic and biochemical update. Nat Rev Cancer.

[10] Brouwers FM, Eisenhofer G, Tao JJ, Kant JA, Adams KT, Linehan WM, Pacak K (2006). High Frequency of SDHB Germline Mutations in Patients with Malignant Catecholamine-Producing Paragangliomas: Implications for Genetic Testing. J Clin Endocrinol Metab.

[11] Amar L, Baudin E, Burnichon N, Peyrard S, Silvera S, Bertherat J, Bertagna X, Schlumberger M, Jeunemaitre X, Gimenez-Roqueplo AP, Plouin PF (2007). Succinate dehydrogenase B gene mutations predict survival in patients with malignant pheochromocytomas or paragangliomas. J Clin Endocrinol Metab.

[12] Jochmanova I, Pacak K (2016). Pheochromocytoma: The First Metabolic Endocrine Cancer. Clin Cancer Res.

[13] Dahia PL (2014). Pheochromocytoma and paraganglioma pathogenesis: learning from genetic heterogeneity. Nat Rev Cancer.

[14] Hsu PP, Sabatini DM (2008). Cancer cell metabolism: Warburg and beyond. Cell.

[15] Hanahan D, Weinberg RA (2011). Hallmarks of cancer: the next generation. Cell.

[16] Tunariu N, Kaye SB, de Souza NM (2012). Functional imaging: what evidence is there for its utility in clinical trials of targeted therapies?. Br J Cancer.

[17] Shankar LK, Hoffman JM, Bacharach S, Graham MM, Karp J, Lammertsma AA, Larson S, Mankoff DA, Siegel BA, Van den Abbeele A, Yap J, Sullivan D (2006). National Cancer Institute. J Nucl Med.

[18] Timmers HJ, Kozupa A, Chen CC, Carrasquillo JA, Ling A, Eisenhofer G, Adams KT, Solis D, Lenders JW, Pacak  K (2007). Superiority of fluorodeoxyglucose positron emission tomography to other functional imaging techniques in the evaluation of metastatic SDHB-associated pheochromocytoma and paraganglioma. J Clin Oncol.

[19] DeNicola GM, Karreth FA, Humpton TJ, Gopinathan A, Wei C, Frese K, Mangal D, Yu KH, Yeo CJ, Calhoun ES, Scrimieri F, Winter JM, Hruban RH, Iacobuzio-Donahue C, Kern SE, Blair IA, Tuveson DA (2011). Oncogene-induced Nrf2 transcription promotes ROS detoxification and tumorigenesis. Nature.

[20] Jaramillo MC, Zhang DD (2013). The emerging role of the Nrf2-Keap1 signaling pathway in cancer. Genes Dev.

[21] Mitsuishi Y, Taguchi K, Kawatani Y, Shibata T, Nukiwa T, Aburatani H, Yamamoto M, Motohashi H (2012). Nrf2 redirects glucose and glutamine into anabolic pathways in metabolic reprogramming. Cancer Cell.

[22] Sporn MB, Liby KT (2012). NRF2 and cancer: the good, the bad and the importance of context. Nat Rev Cancer.

[23] Liu Y, Pang Y, Caisova  V, Ding J, Yu D, Zhou Y, Huynh TT, Ghayee H, Pacak K, Yang C (2020). Targeting NRF2-Governed Glutathione Synthesis for *SDHB*-Mutated Pheochromocytoma and Paraganglioma. Cancers.

[24] Goncalves J, Moog S, Morin A, Gentric G, Müller S, Morrell AP, Kluckova K, Stewart TJ, Andoniadou CL, Lussey-Lepoutre C, Bénit P, Thakker A, Vettore L, Roberts J, Rodriguez R, Mechta-Grigoriou F, Gimenez-Roqueplo AP, Letouzé E, Tennant DA, Favier J (2021). Loss of SDHB Promotes Dysregulated Iron Homeostasis, Oxidative Stress, and Sensitivity to Ascorbate. Cancer Res.

[25] Mizuno T, Kamai T, Abe H, Sakamoto S, Kitajima K, Nishihara D, Yuki H, Kambara T, Betsunoh H, Yashi M, Fukabori Y, Kaji Y, Yoshida K (2015). Clinically significant association between the maximum standardized uptake value on ^18^F-FDG PET and expression of phosphorylated Akt and S6 kinase for prediction of the biological characteristics of renal cell cancer. BMC Cancer.

[26] Kamai T, Higashi S, Murakami S, Arai K, Namatame T, Kijima T, Abe H, Jamiyan T, Ishida K, Shirataki H, Yoshida KI (2021). Cancer Sci.

[27] Casey RT, Ten Hoopen R, Ochoa E, Challis BG, Whitworth J, Smith PS, Martin JE, Clark GR, Rodger F, Maranian M, Allinson K, Madhu B, Roberts T, Campos L, Anstee J, Park SM, Marker A, Watts C, Bulusu VR, Giger OT, Maher ER (2019). SDHC epi-mutation testing in gastrointestinal stromal tumours and related tumours in clinical practice. Sci Rep.

[28] Elliott AM, Radecki J, Moghis B, Li X, Kammesheidt A (2012). Rapid detection of the ACMG/ACOG-recommended 23 CFTR disease-causing mutations using ion torrent semiconductor sequencing. J Biomol Tech.

[29] Xiong L, Xie J, Song C, Liu J, Zheng J, Liu C, Zhang X, Li P, Wang F (2015). The Activation of Nrf2 and Its Downstream Regulated Genes Mediates the Antioxidative Activities of Xueshuan Xinmaining Tablet in Human Umbilical Vein Endothelial Cells. Evid Based Complement Alternat Med.

[30] Favier J, Amar L, Gimenez-Roqueplo AP (2015). Paraganglioma and phaeochromocytoma: from genetics to personalized medicine. Nat Rev Endocrinol.

[31] Dahia PLM, Toledo RA (2020). Recognizing hypoxia in phaeochromocytomas and paragangliomas. Nat Rev Endocrinol.

[32] Jochmanová I, Yang C, Zhuang Z, Pacak K, Hypoxia-Inducible Factor Signaling in Pheochromocytoma (2013). Turning the Rudder in the Right Direction. J Natl Cancer Inst.

[33] Jochmanova I, Pacak K (2018). Genomic Landscape of Pheochromocytoma and Paraganglioma. Trends Cancer.

[34] van Nederveen FH, Gaal J, Favier J, Korpershoek E, Oldenburg RA, de Bruyn EM, Sleddens HF, Derkx P, Rivière J, Dannenberg H, Petri BJ, Komminoth P, Pacak K, Hop WC, Pollard PJ, Mannelli M, Bayley JP, Perren A, Niemann S, Verhofstad AA, de Bruïne AP, Maher ER, Tissier F, Méatchi T, Badoual C, Bertherat J, Amar L, Alataki D, Van Marck E, Ferrau F, François J, de Herder WW, Peeters MP, van Linge A, Lenders JW, Gimenez-Roqueplo AP, de Krijger RR, Dinjens WN (2009). An immunohistochemical procedure to detect patients with paraganglioma and phaeochromocytoma with germline SDHB, SDHC, or SDHD gene mutations: A retrospective and prospective analysis. Lancet Oncol.

[35] Kensler TW, Wakabayashi N, Biswal S (2007). Cell survival responses to environmental stresses via the KEAP1-NRF2-ARE pathway. Ann Rev Pharmacol Toxicol.

[36] Yamaguchi Y, Kamai T, Higashi S, Murakami S, Arai K, Shirataki H, Yoshida KI (2019). Nrf2 gene mutation and single nucleotide polymorphism rs6721961 of the Nrf2 promoter region in renal cell cancer. BMC Cancer.

[37] Nukui A, Narimatsu T, Kambara T, Abe H, Sakamoto S, Yoshida KI, Kamai T (2018). Clinically significant association of elevated expression of nuclear factor E2-related factor 2 expression with higher glucose uptake and progression of upper urinary tract cancer. BMC Cancer.

[38] Chen YB, Brannon AR, Toubaji A, Dudas ME, Won HH, Al-Ahmadie HA, Fine SW, Gopalan A, Frizzell N, Voss MH, Russo P, Berger MF, Tickoo SK, Reuter VE (2014). Hereditary leiomyomatosis and renal cell carcinoma syndrome-associated renal cancer: recognition of the syndrome by pathologic features and the utility of detecting aberrant succination by immunohistochemistry. Am Surg Pathol.

[39] Alderson NL, Wang Y, Blatnik M, Frizzell N, Walla MD, Lyons TJ, Alt N, Carson JA, Nagai R, Thorpe SR, Baynes JW (2006). S-(2-Succinyl)cysteine: a novel chemical modification of tissue proteins by a Krebs cycle intermediate. Arch Biochem Biophys.

[40] Linehan WM, Rouault TA (2013). Molecular pathways: fumarate hydratase-deficient kidney cancer- targeting the Warburg effect in cancer. Clin Cancer Res.

[41] Srinivasan R, Ricketts CJ, Sourbier C, Linehan WM (2015). New Strategies in Renal Cell Carcinoma: Targeting the Genetic and Metabolic Basis of Disease. Clin Cancer Res.

[42] Ooi A, Wong JC, Petillo D, Roossien D, Perrier-Trudova V, Whitten D, Min BW, Tan MH, Zhang Z, Yang XJ, Zhou M, Gardie B, Molinié V, Richard S, Tan PH, Teh BT, Furge KA (2011). An antioxidant response phenotype shared between hereditary and sporadic type 2 papillary renal cell carcinoma. Cancer Cell.

[43] Adam J, Hatipoglu E, O'Flaherty L, Ternette N, Sahgal N, Lockstone H, Baban D, Nye E, Stamp GW, Wolhuter K, Stevens M, Fischer R, Carmeliet P, Maxwell PH, Pugh CW, Frizzell N, Soga T, Kessler BM, El-Bahrawy M, Ratcliffe PJ, Pollard PJ (2011). Renal cyst formation in Fh1-deficient mice is independent of the Hif/Phd pathway: roles for fumarate in KEAP1 succination and Nrf2 signaling. Cancer Cell.

[44] Toledo RA, Burnichon N, Cascon A, Benn DE, Bayley JP, Welander J, Tops CM, Firth H, Dwight T, Ercolino T, Mannelli M, Opocher G, Clifton-Bligh R, Gimm O, Maher ER, Robledo M, Gimenez-Roqueplo AP, Dahia PL, NGS in PPGL (NGSnPPGL) Study Group (2017). Consensus Statement on next-generation-sequencing-based diagnostic testing of hereditary phaeochromocytomas and paragangliomas. Nat Rev Endocrinol.

